# Violent behaviour as a psychopathological symptom: phenomenological distinction from general violence

**DOI:** 10.1192/j.eurpsy.2025.233

**Published:** 2025-08-26

**Authors:** L. Madeira

**Affiliations:** Universidade de Lisboa, Faculdade de Medicina, Lisboa, Portugal

## Abstract

**Abstract:**

Violence is commonly conceptualized as a behavioural act, yet its phenomenological underpinnings reveal significant distinctions between its manifestations in psychopathological conditions and in the general population. This presentation explores how violent behaviour, when emerging as a symptom of psychiatric disorders, differs in its affective structure, intentionality, and embodiment from other forms of aggression. Drawing from phenomenological theories of emotions, we will examine how emotions such as anger, rage, resentment, and hatred manifest differently in psychopathology, particularly in psychotic states, mood disorders, and personality disorders. Emotions are not merely internal states but are lived experiences embedded in bodily spatiality, affecting contraction, expansion, and relationality. In psychopathological contexts, these emotions frequently exhibit disturbances in their anchoring point (what triggers them) and condensation area (where they settle), often leading to dysregulated, disproportionate, or delusionally overdetermined expressions of violence. By contrasting normative anger—typically goal-directed, normatively regulated, and socially embedded—with its pathological counterparts, we uncover crucial distinctions. In conditions such as paranoid psychosis, anger is fused with persecutory delusions, altering its structure from a transient reaction to an entrenched, self-perpetuating stance. Similarly, borderline personality disorder presents dysregulated anger as a core feature, where affective instability fosters reactive aggression that lacks modulation. In psychotic disorders, violent outbursts may emerge in a dissociative or hallucinatory framework, leading to actions detached from conventional interpersonal dynamics. Through a phenomenological analysis, we emphasize how aggressive emotions in psychopathology lack the typical integration of selfhood and social intelligibility, contributing to a distinct kind of violence—one that challenges legal and ethical frameworks regarding responsibility, intentionality, and treatment. By refining our understanding of these differences, we improve both clinical assessment and therapeutic interventions for patients at risk of violent behaviour.

**Disclosure of Interest:**

None Declared

